# Understanding stigma as a barrier to accessing cancer treatment in South Africa: implications for public health campaigns

**DOI:** 10.11604/pamj.2018.29.73.14399

**Published:** 2018-01-24

**Authors:** Tatiana Oystacher, Drew Blasco, Emily He, Debbie Huang, Rebekkah Schear, Devon McGoldrick, Bruce Link, Lawrence Hsin Yang

**Affiliations:** 1 Harris School of Public Policy, University of Chicago, Chicago, United States; 2 College of Global Public Health, New York University, New York, United States; 3 Frances L Hiatt School of Psychology, Clark University, Worcester, United States; 4 Mailman School of Public Health, Columbia University, New York, United States; 5 Livestrong Foundation, Austin, United States; 6 Department of Sociology, University of California, Riverside, United States

**Keywords:** Cancer, South Africa, stigma, knowledge, modified labeling theory

## Abstract

**Introduction:**

Cancer contributes to significant illness burden in South Africa, with delayed diagnosis resulting from limited knowledge of cancer, lack of biomedical treatment and stigma. This study examines ways in which people are identified as having cancer through perspectives of traditional healing or the biomedical model. Additionally, we sought to understand the stigma associated with cancer, including stereotypes, anticipated discrimination and coping styles.

**Methods:**

Livestrong Foundation conducted 11 semi-structured focus groups with key community stakeholders in three South African townships. Interviews examined the negative consequences of being labeled with a cancer diagnosis as well as causes of, possible prevention of and barriers and methods to improve access to cancer treatment. Analyses were completed using directed content analysis.

**Results:**

Revealed three main labeling mechanisms: physical appearance of perceived signs/symptoms of cancer, diagnosis by a traditional healer, or a biomedical diagnosis by a Western physician. Being labeled led to anticipated discrimination in response to prevalent cancer stereotypes. This contributed to delayed treatment, use of traditional healers instead of biomedical treatment and secrecy of symptoms and/or diagnosis. Further, perceptions of cancer were commonly conflated with HIV/TB owing to prior educational campaigns.

**Conclusion:**

Our study deepens the understanding of the cancer labeling process in South Africa and the resulting negative effects of stigma. Future anti-stigma interventions should partner with traditional healers due to their respected community status and consider how previous health interventions may significantly impact current understandings of illness.

## Introduction

**Cancer stigma in South Africa**: Cancer is one of the leading causes of morbidity and mortality globally [1]. In Africa, about 700,000 people are diagnosed with cancer annually with 47,400 cases resulting in death [1]. The American Cancer Society [2] identified that the majority of deferred cancer diagnoses in Africa resulted from limited awareness of early signs of cancer, lack of biomedical screening and stigma. Cancer stigma often originates from cultural stereotypes that cancer is a fatal disease, is contagious, or is a punishment for immoral behavior [3, 4]. Additionally, cancer stigma has received less attention than communicable disease stigma (e.g. HIV/AIDS) [5]. Further, severe stigma associated with cancer has likely contributed to delays in biomedical treatment [5]. Social consequences of being labeled with a cancer diagnosis (e.g. shame) may deter use of biomedical screening, resulting in treatment delays [6-8]. Public education campaigns to increase knowledge and decrease stigma are crucial in promoting prevention and treatment of cancer in South Africa [9, 10]. Implementing interventions in culturally sensitive ways can enhance the reach of educational messages [11, 12] and increase adherence with treatment [13]. Additionally, successful strategies may include partnering with traditional healers. This has been used to increase diffusion of information regarding HIV/AIDS awareness. In South Africa, estimates range from 60% of people consulting traditional healers [14] to approximately 50% consulting both biomedical providers and traditional healers [15]. South African healers are critical advisors in the use of healthcare in their communities [16-21]. Understanding misconceptions about cancer and best practices to deliver culturally-appropriate educational messages is crucial for successful implementation of educational campaigns. This study investigates cancer stigma as a barrier to help-seeking within a South African community. Additionally, we seek to address a gap regarding knowledge of cultural stereotypes around cancer, common stigmatizing conceptions and range of discriminatory experiences. Our findings may inform interventions aimed to raise awareness, increase biomedical screenings and treatment for those diagnosed with cancer.

**Study and theoretical framework**: This paper presents data collected from 2010-2012 in three South African towns prior to Livestrong Foundation's public education efforts to reduce cancer stigma. We utilize Link and Phelan's [22] “Modified Labeling Theory” as a framework to understand local cancer stereotypes and mechanisms in which stigma manifests. We utilized three concepts (i.e. stereotyping, anticipated discrimination and coping mechanisms) from this framework to understand how individuals are stigmatized and excluded from everyday interactions. Primarily, Link and Phelan's framework has been used to examine mental illness stigma and applied to conditions of HIV [23, 24], obesity [25, 26] and hepatitis C [27]. We propose extending this theory to the illness condition of cancer, which has been relatively understudied [23]. We explore differences in how South African respondents may use local understandings of cancer in contrast to the biomedical model. We also examine ways in which stigma may manifest through different labeling processes as applied to cancer. For example, labeling may occur through formal Western diagnoses, diagnosis by traditional healers, or showing cancer symptoms. Given the prominence of traditional healing in South Africa, we seek to understand how traditional medical practices may be connected to stereotypes and stigma toward cancer and how avoidance of Western treatment labeling may influence use of treatment services. Additionally, we seek to illuminate predominant coping strategies arising from a cancer diagnosis such as withdrawal, secrecy, or how anticipating discrimination may deter someone from biomedical treatment. Finally, we utilize a rare opportunity to analyze the unexamined connection between HIV and cancer stigma in South Africa as a potential effect of prior national stigma reduction efforts for HIV [28-32].

## Methods

**Study design**: The livestrong foundation interviewed more than 4,500 healthcare providers, cancer survivors, NGOs and experts across 10 countries. Results compelled the Livestrong Foundation to conduct a pilot anti-stigma campaign in South Africa from 2010-2012. Prior to this cancer education campaign, Livestrong Foundation conducted a series of qualitative interviews and focus groups with key community stakeholders to understand cultural conceptions of cancer stigma which we report upon here.

**Data collection**: Eleven semi-structured focus groups were conducted in three peri-urban townships within South Africa: Khayelitsha, Soweto, and East London. Townships held focus groups consisting of: traditional healers (3 groups, n = 21 participants); church groups (3 groups, n = 29 participants), non-government organizations (NGOs) (2 groups, n = 33 participants); community leaders, including political leaders and members of community health forums (3 groups, n = 37 participants). Age range of church leaders and traditional healers was 55-75 years, while the age range in other groups was 26-65 years. To encourage open disclosure concerning potentially highly stigmatizing personal, family, or community experiences of stigma, no other personal information was collected. Semi-structured interviews focused on the following: causes of and possible ways of preventing cancer, contact with someone diagnosed with cancer and treatment of cancer patients by the community. In addition, focus group participants discussed how one would be treated and what a person would do/feel if he/she was diagnosed with cancer. Other topics queried included: the role of media in disseminating cancer knowledge, barriers to help-seeking and how access to prevention and treatment might be improved. Follow-up questions were asked as appropriate. All focus group interviews were audio-recorded and transcribed by South African transcription services. Data analysis was approved by the Columbia University Medical Center Institutional Review Board.

**Data analysis**: We based our analytic scheme on Link and Phelan's [21] multidimensional model of stigma. We examined ways cancer stigma might cohere and differ, with key stigma components (i.e. labeling, anticipated discrimination and coping mechanism), which have been predictive of negative outcomes [33-36]. Using directed content analysis [37], we sought to validate and extend conceptual frameworks for cancer-related stigma. Following Link and Phelan's framework as a guide, we first determined analytical schema and initial coding categories. Three research team members (T.O, D.H, E.H) and the principle investigator (L.Y.) coded the 11 focus group transcripts using the initial coding scheme. First, one transcript of each respondent type (traditional healers, NGO's, community leaders) was group-coded to modify initial coding categories. After establishing consensus, each remaining transcript was coded independently by all research team members and any discrepancies discussed. Initial coding categories were modified based on discovery of conceptual relationships between codes (e.g, merging subcodes that represented different aspects of a single coding category). While following a deductive framework, the codebook was expanded inductively to account for new concepts which arose due to culturally-specific experiences of cancer in South Africa. Discrepancies in codes were resolved during consensus meetings. The final codebook is shown Annex 1. Transcripts were analyzed using ATLAS-TI.

## Results

**General cancer knowledge and stereotypes**: Respondents' cancer knowledge varied from no previous knowledge to some knowledge of risk factors, etiology, treatment and prognosis. Respondents cited knowledge of common cancers and risk factors (i.e smoking, drinking, unhealthy lifestyles and excessive sun exposure). Multiple stakeholders characterized cancer as deadly and incurable, leading to fear of diagnosis, hopelessness and depression (Table 1). However, some respondents reported cancer being curable in the early stages. They named methods of medical treatment-“being ironed” (getting chemotherapy) or “ being burned” (getting radiotherapy)-and accompanying side effects, such as nausea, difficulty eating and hair loss. Yet multiple groups of respondents revealed uncertainty about causes, transmission and treatment (Table 1). Respondents identified four prominent stereotypical causes for cancer: “physical contagion”, “witchcraft”, “influence of the culture of White colonizers” (i.e, adopting their diets) and “sexuality/promiscuity”. This last cause included early pregnancy, multiple childbirths and even breastfeeding (Table 1). These prominent stereotypes often led to anticipated discrimination.

**Table 1:** Knowledge and stereotypes of cancer

Knowledge - Prognosis of cancer (deadly and incurable)	
1.	“We were also told that he was going to die, and we just watched him going through the agony.” (Soweto NGO group)
2.	“I would think of committing suicide… I would be shocked if I’m told it is cancer I have.” (Soweto Church group)
3.	“I would be stressed and depressed every day. I would always be sick because of stress.” (Soweto Church group)
**Knowledge - Uncertainty about causes of cancer**	
4.	“What about a person who does not smoke and still gets cancer? Because my mother did not smoke and she did not drink.”(East London Traditional Healers’ group)
5.	“I want to know what causes these cancers.” (Khayelitsha Church group)
6.	“Another thing is that I don’t understand is how one can inherit cancer. How can one get something from someone [without physical contact] and [without having similar] lifestyle?” (Soweto Political and Community Leaders’ group).
**Stereotype - Physical Contagion**	
7.	“…giving you a different bed, blankets and dishes to use” (Khayelitsha Church group);
8.	“Even if you bathe him, you put on a mask” (East London Church group).
9.	“When you help by trying to put herbs on the wound, you may touch and thereby get cancer” (Soweto Traditional Healers’ group).
**Stereotype - Witchcraft**	
10.	“We black people have this belief that there are sicknesses that come as a result of witchcraft… Even if one is told that its cancer they may not even believe it…” (Soweto Political and Community Leaders’ group).
11.	“The reason why we believe that it is witchcraft is because some people that are affected will mention that they were poisoned. In the black communities we believe that it is sold, and people can buy it to give it to other people” (Khayelitsha Traditional Healers’ group).
12.	“People only know about oesophagus cancer and because they do not know what the course is and thought they were bewitched.”
13.	“People can put cancer in your food, those who hate you. But when you have God, you forget who is bewitching you, you just focus on yourself.” (East London Church group)
**Stereotypes attributed to White group**	
14.	“Even children get cancer; they get it when they are still very young, especially white people.” (East London Traditional Healers’ group)
15.	“I have been thinking that cancer is for white people and a lot of people think like that.” (Soweto Church group)
16.	“They call cancer a white man disease because white people eat refined food. We, black, people get it a lot because we have adopted the white man’s food. Research has shown that what black people used to eat did not promote the abnormal growth of cells as in cancer.” (Soweto Traditional Healers’ group)
**Stereotypes - Gender, Sexuality, Promiscuity**	
17.	“I used to be promiscuous with boys, so I think I can get it (cancer).” (Soweto NGO group)
18.	“Also, because I got pregnant at an early stage … so I think I can get it (cancer).” (Soweto NGO group)
19.	“Because I have many children I think I can have it.” (East London NGO group)
20.	“Yes, I can have it, because I am breastfeeding, so I may have it because of that, breast cancer.” (East London Church group)

**Anticipated discrimination**: From these stereotypes, the most prominent topic concerned anticipated discrimination/devaluation. In addition to providing examples of discrimination against people with cancer by community members, respondents also predicted that they themselves would discriminate against their relatives/partners with cancer (Table 2).

**Table 2:** Devaluation and discrimination

Discrimination stemming from the belief in contagion	
1	“…if someone come for help and they are wounded… When you help by trying to put herbs on the wound, you may touch and thereby get cancer.” (Soweto Traditional Healers’ group)
2.	“The thing is that whenever you treat someone you must use hand gloves” (Soweto Traditional Healers’ group).
3.	“People avoid people with cancer for fear of being infected” (Khayelitsha Church group)
4.	“I think that would depend on how your family treats you, people will look at how you are being treated by your own family, if they treat you badly like giving you a different bed, blankets and dishes to use then the people from the outside will learn from that and treat you the same way” (Khayelitsha Church group).
**Discrimination stemming from beliefs about sexuality and promiscuity**	
5.	“…If you get prostate gland cancer then you got [it] from immoral behavior…” (Soweto Political and Community Leaders’ group).
6.	“People with cervical cancer are the ones that get discriminated against a lot because people associated it with sleeping around.... With cervical cancer it is like one has got what they deserved.” (Soweto NGO group)
7.	“When I have cancer and my partner runs away, it can happen that he also has it, he may have contracted it from me.” (Khayelitsha Church group)
**Discrimination stemming from beliefs in witchcraft**	
8.	“…It may be the neighbour that has bewitched the person and you will be giving them joy by letting them know that you have cancer” (Khayelitsha Church group).
**Forms of Discrimination**	
9.	“I prefer to use myself as an example, if my partner was to be HIV positive which is similar to cancer, after years of being together it would be difficult for me to accept my partner.” (Khayelitsha Church group).
10.	“They will stop calling you even if they used to call you, because they are worried thinking that you are going to be a burden to them.” (East London Traditional Healers’ group).
11.	“People change even within the family as they do not want the responsibility of having to care for someone who is sick. Even children do not want to take care of their sick parents” (East London Traditional Healers’ group).
12.	“She always complained that ever since people heard that she had cancer they did not come to her house.” (East London Traditional Healers’ group).
13.	“I know one person whose husband left her because she had cancer” (Soweto Traditional Healers’ group).
14.	“I know of someone who had cancer and stayed with a girlfriend who disappeared after hearing that he had cancer” (Soweto Traditional Healers’ group).
15.	“It really hurts, to see somebody in that situation. People are leaving their families because of this thing.” (East London Church group)
16.	“I think that a person with cancer is not treated well, we don’t treat them well, and we isolate him.” (East London Church group)
17.	“I have an example of a neighbor who had children who ran away and did not want to support him when he was diagnosed with cancer.” (Soweto Church group).
18.	“Once you have cancer, it’s like when you have AIDS people isolate you, and it eats you up…” (East London Political and Community Leaders’ group).

**Cancer linked to physical contagion, sexuality and promiscuity and witchcraft**: The belief that cancer is contagious was a source of anticipated discrimination and isolation. Traditional healers and a church respondent discussed fear of contracting cancer through contact with cancer patients (Table 2). This belief resulted in avoidance of sharing items, proving separate bedding and tableware (Table 2). Notably, how a person was treated by their family could influence the attitudes of community members (Table 2). Multiple stakeholder groups endorsed stereotypes that cancer was caused by sexual promiscuity, was linked with cancer of reproductive areas and was acknowledged as punishment for immoral behavior (Table 2). Stereotypes that cancer may be sexually transmitted caused significant shame and justified ending a relationship. Finally, witchcraft stereotypes were attributable to the cancer resulting from a “curse” and someone wishing harm to a “bewitched” person (Table 2). Regardless of stereotype, cancer patients experienced a range of discriminatory occurrences from being perceived as burdensome (Table 2) to being isolated from both their family and broader social network (Table 2). Participants feared they would face severe forms of social discrimination from being labeled as a cancer patient.

**Mechanisms of Labeling**: Analyses indicated ways in which a person acquired the label of a “cancer patient”, some of which were more definitive than others: 1) labeling through appearance of perceived signs or symptoms of cancer, or treatment side effects; 2) cancer diagnosis from a traditional healer; 3) cancer diagnosis by a western physician.

**Signs and symptoms of cancer or effects of cancer treatment**: Respondents named signs and symptoms leading to identification of a person as having cancer in the absence of a medical diagnosis. These included lumps on different parts of the body (including “abnormal things on private parts”), “problems with the throat” (e.g, difficulties swallowing, stabbing pain in the throat, coughing and having a lot of phlegm), visible weight loss and energy loss. Respondents also mentioned back pains and unpleasant odor coming from affected individuals. Further, participants identified cancer signs related to side effects of Western treatments, such as temperature changes, nausea, hair loss and in the case of breast cancer, extirpation of a breast (Table 3).

**Table 3:** Labeling process

Side effects of treatment	
1.	“I also met someone yesterday, who takes cancer treatment and he had nausea and vomiting and was weak. A doctor told him that the treatment was causing that.” (East London Church group)
2.	“I also hear about the brain, the hair sometimes fall off because of it.” (Khayelitsha Church Group)
3.	“Even when people saw children on TV without hair they would have no idea what is wrong. After it affected a celebrity people got to know about it.” (Soweto NGO group)
4.	“…they would be disturbed, thinking about the hair, that when you comb it, it will fall off.” (East London Political and Community Leaders’ group)
5	“I got information from a friend that a person must not dump his wife because her breasts are cut off, that’s how he told me.” (East London Church Group)
**Diagnosis by traditional healer**	
6.	“People first come to us as traditional healers before going to the doctors and clinics…” (East London Traditional Healers’ group)
7.	“With us, people just come any time; we do not have a practice that opens at certain times like 8am to 6pm” (Soweto Traditional Healers’ group).
8.	“The first thing I would do as the person who believes in traditional medicine, I would go to a traditional person who would help me.” (East London Traditional Healers’ group)
9.	“When we grew up cancer was a White people disease. We used to eat wild fruits and roots and never used to get sick… We as traditional healers use herbs that are organic to treat” (Khayelitsha Traditional Healers’ group)
10.	“People never used to die so much in the olden days when they were using traditional medicines” (Khayelitsha Traditional Healers’ group)
11.	“Some come having been diagnosed already by the doctors but are scared of operations and opt for traditional medicine” (Khayelitsha Traditional Healers’ group)
12.	“We black people have this belief that there are sicknesses that come as a result of witchcraft. You find someone who will not seek medical help early enough because they were told that they have been bewitched. I have a relative who has been sick for a long time and has been going to sangomas who tell her she has been bewitched, but this year she was diagnosed with cancer.” (Soweto Political and Community Leaders’ group)
13.	“We don’t have the x-rays to see where the cancer is hence we send them to the doctor.” (East London Traditional Healers’ group).
14.	“If someone comes to me [traditional healer] complaining of pain, I will tell them to go to the doctors who can examine them using sophisticated machinery before I can try and treat them” (Soweto Traditional Healers’ group).
**Official biomedical diagnosis**	
15.	“You may have just taken your sister to a white doctor and you may not understand any word except the word cancer. (Soweto Political and Community Leaders’ group).
16.	“It is true that people do not go to the clinic. I think there should be a group going door to door (to detect individuals with cancer) because… some (people) are scared of being diagnosed.” (East London church group)
17.	“I had something in my throat, it started off like something like a ball, when I swallowed it would feel like it is going down. I went to the clinic and I was given tablets that I don’t know what they were. I never went to the doctor again, but I still feel it because I am very scared to go to the doctor. I don’t know whether it is cancer or what.” (Khayelitsha Political and Community Leaders group)

**Diagnosis by traditional healers**: According to traditional healers' perspectives, they are frequently utilized as healthcare providers first because of their easy access in communities (Table 3) and their perceived efficacy (Table 3), especially when cancer is believed to be caused by witchcraft (Table 3). Use of traditional healers may enable avoidance of the perceived invasiveness of Western cancer treatment (Table 3). While dissenting views existed, cancer diagnosis “labeling” was not as definitive when provided by a traditional healer. Instead, some traditional healer respondents reported that they could not provide a formal cancer diagnosis, thereby referring individuals to western doctors for diagnosis using biotechnology (Table 3).

**Biomedical diagnosis**: The most definitive “labeling” method was receiving a diagnosis from a western physician. This labeling process was initiated by cancer screenings at the clinic and culminated in receiving a biomedical diagnosis. While initiating beneficial biomedical treatment, this label also elicited the above cancer stereotypes. Multiple stakeholders cited that an official biomedical diagnosis (and biomedical treatment) was associated with fear, avoidance of treatment and rejection from others (Table 3).

**Coping mechanisms in response to labeling and anticipated discrimination**: Several coping mechanisms, or ways those “labeled” as having cancer responded to their stigmatized status, were identified. Secrecy was the most common response. Participants provided examples of cancer patients refraining from disclosing the disease to their families or community until it was terminal. Multiple stakeholder groups cited that secrecy from family members was attributable to lack of knowledge of the signs/symptoms of cancer, fear of death, shame, concern about being laughed at or feared and fear of witchcraft (Table 4). Additionally, participants reported individuals with cancer might be afraid of stigma and discrimination from their families, spouses and community (Table 4). Less frequently mentioned were negative coping strategies of denial or withdrawal (i.e. not performing checkups due to not knowing what might be discovered or of being diagnosed with what is perceived as an incurable disease) (Table 4). Respondents openly discussed whether cancer may be treatable during its early stages. Additionally, they identified that being secretive and not seeking prompt treatment may lead to worse health outcomes. Less frequently, multiple stakeholder groups suggested openness, acceptance and self-education as positive ways of coping with a cancer diagnosis (Table 4). While less common, these provide examples of more effective coping strategies that might be built upon for future interventions.

**Table 4:** coping mechanisms and emotional response

Secrecy	
1.	“…my aunt had cancer. It started as a small mark around the groin. She was reluctant to disclose what was wrong with her until she was on her death bed” (Soweto Traditional Healers’ group).
2.	“The reason why people with cancer hide it it’s because they do not know much about cancer, about what it is” (East London Traditional Healers’ group).
3.	“…people are afraid to talk about it is that people are scared to die or for their families to know that they are dying” (East London Traditional Healers’ group).
4.	“People are embarrassed to say they have cancer. They hide it when the disease is spreading (in their bodies).” (Khayelitsha Traditional Healers’ group)
5.	“I had three people at home who had cancer. My brother had it on his private parts, his wife on her breast and my aunt on the breast. They all hid it because they were scared of people laughing at them” (East London Church group). “
6.	“I would be scared to tell people about cancer because people will fear me, like a person with AIDS.” (Khayelitsha Church group)
7.	“Families **hide people** because they believe that they are bewitched and cannot be helped anywhere.” (East London NGO group).
**Denial and withdrawal**	
8.	“People with cancer come out when it is already worse, and it is in its terminal stage because they don’t know.” (Khayelitsha Church group)
9.	“…its few people who respond to these messages, I can say maybe 20% of women go for pap smear, some are afraid of the unknown” (East London Traditional Healers’ group).
10.	“…people do not go (to seek early detection screening) because they do not want to know that they are dying” (East London Traditional Healers’ group).
11.	“…even if you cough a lot it’s hard to go and test for it because you are scared they will say you have cancer, I’m running away from it.” (East London Political and Community Leaders’ group).
**Positive coping strategies**	
12.	If the doctor has told you that you have such a disease, you need to accept and tell your family, telling will help you to heal. (Khayelitsha Church group)
13.	People have to first accept themselves before they expect to be accepted. It is important to have hope for the future before even after being diagnosed with cancer. (Soweto Political and Community Leaders’ group)
14.	It will depend on the type of person that you are, you should share with your close friends and family, tell them that you are in this kind of a trouble, but help can be helped, you should educate them. (East London Political and Community Leaders’ group)

**Stigma's impact on accessing treatment**: Multiple stakeholder groups revealed distrust toward western health services or a lack of understanding of biomedical procedures, leading to fear. Several traditional healers and church leaders blamed western medical epistemology for misdiagnosis, inability to help and even for unethical experimentation on Black people. Additionally, NGO and community leader groups expressed concern that western medical professionals were not providing sufficient accurate information to communities (Table 5). Although direct stigma perpetuated by individual health providers and/or organizations of power (e.g. employers, government agencies, hospitals) were not observed, these commonly-held perspectives portrayed Western modes of treatment and of understanding disease, as an extension of an oppressive, colonialist government. This not only had negative consequences for early detection and treatment but also was seen as a tool to suppress the Black population (Table 5). The lack of consistency about the role of traditional healers in cancer treatment contributed to a disconnect between traditional healing and Western medicine. Therefore, even when traditional healers were utilized, patients might not be referred to western biomedical treatment. NGO groups and church healers held more pessimistic views towards traditional healing. NGO groups cited traditional healers as being detrimental to public health. Church leaders spoke about their belief that information provided by traditional healers often delayed Western medical diagnosis (Table 5). Community leaders rarely expressed negative views of traditional medicine and recognized the frequent use of it as a response to lack of biomedical information or mistrust of Western medicine (Table 5). Traditional healers saw themselves as valuable partners in the cancer-treatment process due to their accessibility in the local community and ability to facilitate referrals. Some of them expressed the desire to augment their own knowledge with Western medical knowledge. However, traditional healers were also concerned that Western providers would not cross-refer to them and that they were not recognized as legitimate by the biomedical field (Table 5).

**Table 5:** services and treatment

Lack of understanding and distrust towards biomedical procedures	
1.	“My brother had it on his private parts, his wife on her breast and my aunt on the breast. They all hid it because they were scared of people laughing at them. They used traditional medicine until it was late, and nothing could be done and later died.” (East London Church group)
2.	“What I noticed is that most people don’t know much about cancer, so I wish we can get an understanding of what it is. People think that it’s a curable disease, they go to witchdoctors and they don’t like going to the doctors, they say that if you go to the doctors you will die.” (East London Political and Community Leaders’ group)
3	“While he was in hospital his mother kept on bringing him traditional medicine, because people don’t believe that the drug given at hospital can help, they believe that the drugs at the hospital are weaker that the traditional. I agree with the other speakers that people do not have clear information on cancer. People end up seeking help from traditional practitioners because doctors cannot cure cancer.” (East London Political and Community Leaders’ group).
4.	“No, I don’t think so, I will talk about the area that I’m living in, if someone has cancer, they only think of (going to see) witchdoctors.” (East London Political and Community Leaders’ group)
5.	“I think the problem that we all have is relying too much on the (Western) doctors who will tell you to take treatment. Sometimes the doctors themselves prescribe incorrect treatment and or dosage. They sometimes misdiagnose you, telling you that you have such a disease and you stress until you become thin, only to find out there is not such. The doctor said to me that I have cancer, but I did not have cancer. I was treating cancer all the time only to find out later that it was not cancer” (East London Church Group).
**Fear of unethical experimentation and suppression of Black population**	
6.	“Three weeks ago I was burying my father who died from prostate cancer. He was admitted several times at the hospital. I kept warning him that they will kill you in hospital as they experiment with black people.” (Soweto Traditional Healers’ group)
7.	“My view is that this is colonialism to suppress a black man not to know. My personal view is that AIDS and cancer is orchestrated by some people in America to ensure that our people die. It is aimed at destroying the kids, the future of blacks particularly. The issue is they bring terminologies we are not familiar with knowing very well that our people are not educated to a level suppose so that they will be able to understand what is being said. It is a way in which white people are trying to reduce the population otherwise they lose the vote” (Soweto Political and Community Leaders’ group).
8.	“The warnings on tobacco are written by white people so I do not agree with this western culture.” (East London Traditional Healers’ group).
**Discrediting of Traditional Healing by Western Medicine and Other Stakeholders**	
9.	“And the way they explain it, it makes a person who has any other cancer besides breast and cervical cancer not to go for medical help early enough when there is something wrong with them and not being the breast or cervix. They end up trying traditional medicine sometimes [which is] not helpful instead of going to see the doctor” (Soweto Political and Community Leaders’ group).
10.	“Correct information should be given to people as they use traditional healers, they give irrelevant information and by the time you go to a doctor it is late because cancer spreads in the body.” (Soweto Church group)
11.	“People are dying of this because they are not getting help from the witchdoctors…” (East London Political and Community Leaders’ group).
12.	“We as traditional healers need to be recognized by medical doctors. We need to be able to get referrals of patients from them as we also refer to them” (East London Traditional Healers’ group).
13.	“**We as traditional healers are portrayed badly to the community** and as a result it looks like **our services are stigmatized where as we want recognition from the western medicine**” (East London Traditional Healers’ group).
14.	“We can also educate people as we were also trained by the Department of Health. **Cross referral can also happen**” (East London Traditional Healers’ group).

**HIV/TB education campaigns and future cancer campaigns**: Past education campaigns such as with HIV/AIDS and TB made promoting education and reducing stigma around cancer more complex. Participants identified the success achieved by involving multiple community and healthcare organizations in HIV and TB awareness campaigns, thus laying a foundation for cancer education. First, multiple stakeholder groups stated that previous campaigns established methods of outreach including community screening (Table 6), workshops (Table 6) and involving churches (Table 6) and youth in community education (Table 6). Second, members of church groups cited valuable outreach strategies including nurses entering communities to provide education regarding symptoms and check-ups (Table 6) and implementing testing for both HIV and cancer (Table 6). Third, prior campaigns have made significant efforts to access hard-to-reach populations by using appropriate idioms understood by the community (Table 6). Finally, delivery of an educational and anti-stigma campaign for cancer could be modeled after prior HIV campaigns, which have focused on showing real people successfully fighting HIV (Table 6).

**Table 6:** interrelation between beliefs about cancer and HIV

1.	“With TB for example, there were many people going around Khayelitsha testing people, they can do the same for cancer. I mean even to cancer they can do that because they test TB, HIV, why not Cancer…” (Khayelitsha Church group)
2.	“If people can be called to places like stadiums, as they do with TB and other diseases, then a certain area like Mdantsane can go and hear about cancer.” (East London Political and Community Leaders’ group).
3.	“Holding workshops, preaching about it in churches. We need to do as much as we have done about HIV.” (Soweto Church group)
4.	“Churches do have organizations whereby they work together like the Council of Churches they can work together. They have done it with HIV where they have educating people about HIV.” (Soweto Church group)
5.	“Getting the youth involved that are going to educate the community using entertainment. If it happens in HIV, why not in cancer.” (Soweto NGO group)
6.	“If nurses can reach out to the people at community level, definitely people can survive cancer... People do not take wounds seriously. People do not go for follow ups and nobody follows up on them.” (Khayelitsha Church group)
7.	“The JSI project must organize nurses who will come and do check-ups every three months or so. That will give people an opportunity to check themselves.” (East London Church group)
8.	“If there can be some kind of a joint venture between the cancer campaign and HIV & AIDS campaign in order to ensure that when a person tests for AIDS is able to test for cancer, for early detection and also preventive measures to be put in place. I think it would help.” (East London Church group)
9.	“For us black people if you tell the old generation in the rural areas about cancer, they will not understand. Just like with HIV, they had to invent an African name for it, i.e. Ingculaza (AIDS) if you come to us telling us about cancer, what is cancer to the elderly – remember many of us are not educated.” (Soweto Political and Community Leaders’ group).
10.	“I think we can learn something from the HIV & AIDS campaign. When they started the campaigns, they used frightening ads but that did not help. When they changed their ads to a story telling kind and put characters there real-life characters who look like ordinary people. Those messages were able to hit home. People are no longer afraid to test for HIV now which is more deadly than cancer, but not cancer diagnosed in the later stages because it can be as deadly.” (Soweto Political and Community Leaders’ group).
11.	“…Some of them (people with cancer) have symptoms that are similar to those of HIV and because we do not know about the symptoms of cancer, so they mistake the two” (Soweto NGO group).
12.	“I can’t differentiate personally what is AIDS and cancer” (Soweto Political and Community Leaders’ group).
13.	“One can get STI and they progress to HIV and from that it progresses to cancer” (Soweto NGO group).
14.	“Families do not disclose - people think about death, it is associated with HIV” (East London NGO group)
15.	“if my partner was to be HIV positive which is similar to cancer, after years of being together it would be difficult for me to accept my partner.” (Khayelitsha Church group)
16.	“Now things are better but because people do not know about signs of cancer, so they isolate a person with cancer, but because stigma for HIV as well has subsided so things are not that bad.” (Soweto NGO group).
17.	“We always hear only about TB and HIV.” (Khayelitsha Traditional Healers’ group)
18.	“Community Health care workers here are only doing TB and HIV and AIDS education. There is nothing about cancer… Even support groups here are for HIV and TB you never hear about cancer. They do talk about it a lot on the radios and TV but not enough.” (Khayelitsha Traditional Healers’ group)
19.	“The focus is on HIV and AIDS. People do not know about cancer and there is no one to teach them.” (Soweto Church group)
20.	“Because people with cancer hide themselves, there were many people with cancer in the past but because of HIV fewer people are known”. (East London NGO group)
21.	“Even nurses do not talk about cancer, what they always talk about is AIDS and TB and not cancer, some people don’t even know the symptoms.” (East London Political and Community Leaders’ group).

**Conflation of beliefs about cancer with beliefs about HIV/TB**: The after effect of prior campaigns also appeared to have an unintended consequence of conflation of HIV/AIDS and TB with cancer among community members. Multiple stakeholder groups were unable to differentiate between the symptoms of HIV/AIDS and cancer (Table 6). Multiple respondents stated that because a cancer diagnosis is perceived by the community as eliciting the same deadliness as HIV/AIDS, a cancer diagnosis may lead to comparable negative responses, including isolation and abandonment (Table 6). However, comparisons with HIV/AIDS may also be beneficial. An NGO group respondent stated that reducing stigma for HIV contributed to reducing cancer stigma (Table 6). Multiple stakeholder groups, however, perceived structural discrimination against providing cancer education due to the healthcare system's focus on information and access to HIV/AIDS and TB prevention and treatment (Table 6).

**Use of media**: Media outreach, including television, radio, or newspapers, was identified as a central way to educate people about cancer. Multiple stakeholder groups emphasized stories of real people and celebrities who spoke about their experience fighting cancer (Annex 2). However, factual information about causes, symptoms, course and treatment methods were lacking (Annex 2). Multiple stakeholder groups cited that previous HIV educational programs had successfully used community outreach through local churches, schools, and traditional healers, which could be leveraged for providing biomedical information and antistigmatizing messages about cancer ( **Annex 2** ).

## Discussion

Our study identifies three distinct ways community members may be labeled with a cancer diagnosis. We further identify the resulting anticipated discrimination and negative coping mechanisms employed, including secrecy. Our study builds on previous research illustrating the negative consequences of being labeled, including delays in accessing biomedical treatment in South Africa [4, 7, 8]. Respondents named a variety of signs and symptoms perceived to be caused by cancer leading to a cancer label, even in the absence of a biomedical diagnosis. Generally, while respondents agreed that diagnosis by a biomedical physician was the most definitive owing to biotechnology, this label also elicited fear due to negative stereotypes and potential rejection by other community members. Participants often deemed a diagnosis provided by a traditional healer as less conclusive. Traditional healers were often utilized by community members due to etiological beliefs of witchcraft, their accessibility, their perceived effectiveness in treating cancer and the lack of invasiveness of traditional treatment. Further, we found evidence that negative stereotypes corresponded with anticipated discrimination and the desire to be secretive regarding one's diagnosis. Although these links were not explicitly stated, these findings are consistent with empirical studies of Modified Labeling Theory [33, 34, 36], which demonstrate that these processes are interlinked. Finally, this study highlights an unexplored finding; namely, that previous HIV interventions may unintentionally act as a significant source of etiological misunderstanding and an additional source of stigma in South African communities (see below).

Because of the stigma elicited by a biomedical diagnosis of cancer and mistrust of Western hospitals, a powerful theme emerged whereby some respondents indicated a preference for traditional healing. While dissenting views existed, some community members believed in the efficacy of traditional healers to treat cancer. Our results are generally consistent with studies showing that traditional healers are utilized first due to their accessibility [13-21]. Future education campaigns might involve traditional healers in the process of identifying those who may benefit from cancer treatment, and may act as key referral sources for biomedical practitioners. To capitalize on some traditional healers' interest in increasing knowledge of Western medicine, we propose that partnerships between traditional healers and Western physicians may increase referrals to Western biomedical treatment. Developing these partnerships may also allow space for cultural interpretations of illness. Future studies might also examine whether integration of traditional healers into medical services is associated with greater access to treatment, and less stigma. Due in part to the prior implementation of educational campaigns to address HIV, multiple stakeholder groups conflated symptomatology of HIV with cancer symptoms. Our findings build on a prior study where confusion between HIV symptoms and diabetes was reported among comorbid patients in Cameroon [13]. In our study sample, increased stigma appeared to result from beliefs concerning the interconnectedness of HIV/AIDS and cancer, the inability of participants to differentiate between these illnesses and the relative lower institutional priority given to cancer education. Additional cancer stigma due to conflation of stereotypes of deadliness associated with HIV may contribute to negative consequences, including social isolation. Yet positive aspects of prior HIV/AIDS education campaigns also emerged. Due to the perceived similarity of these two illnesses, instances of stigma reduction around HIV/AIDS sometimes also led to reduction of stigma for cancer.

Additionally, multiple stakeholder groups cited successful approaches for community outreach for future cancer campaigns, including community screening, workshops and involvement of churches and youth in educating communities. Other than the prior cited study [13], few studies to our knowledge have examined the effects of campaigns following prior educational efforts. To maximize efficacy, future campaigns should take into consideration: a) prior outreach methods; b) preexisting knowledge of other illnesses; c) and how education around new illnesses might interact with preexisting knowledge. Our findings should be considered in light of several limitations. First, data collection occurred in a small community in South Africa and thus cannot be generalized outside this context. Additionally, to protect participants' confidentiality and reduce the possibility of social desirability when inquiring about stigma, limited demographic information was collected. Finally, interviews were conducted at one time point prior to the implementation of the stigma education campaign; we are therefore unable to examine potential changes in stigma during the campaign. This study has several strengths, including the use of qualitative methodology to more thoroughly understand the nuanced nature of stigma. We recruited a sample of diverse stakeholders including traditional healers, church groups, NGOs, community and political leaders and members of community health forums, allowing for a broader sampling of perspectives. Further, to our knowledge, excluding studies examining this issue in connection to cervical cancer [4, 7, 8, 11], this is one of the first studies to examine cancer stigma in South Africa. Finally, this study draws on Link and colleagues Modified Labeling Theory and thus we propose the possible extension of this framework to a new illness condition.

## Conclusion

In conclusion, our study advances the literature by demonstrating the significant negative effects of cancer stigma in South Africa. Using Link and colleagues Modified Labeling Theory, we identified three distinct ways individuals may be labeled with a cancer diagnosis. Consideration of these three labeling mechanisms and their resulting outcomes is important when designing future educational interventions. Future campaigns should examine the possibility of including traditional healers as key collaborators for cancer treatment to increase community member referrals, with the ultimate goal of increasing uptake of biomedical treatment. Finally, when designing new community education campaigns, the potential impact of previous campaigns should be carefully considered.

### What is known about this topic

- Cancer is a significant cause of morbidity and mortality in Africa;
- Stigma of cancer and the social consequences of being labeled as a cancer patient are causes of delayed diagnosis and treatment;
- There have been limited studies in South Africa on cancer stigma; however, prior studies of HPV and Pap-smear awareness campaigns have indicated stigmatizing views toward individuals with cancer.

### What this study adds

- An in-depth understanding of the three most prevalent ways in which one may be labeled with a cancer diagnosis in the community, and the consequences of stigma that result from: 1) biomedical diagnosis via a Western physician; 2) appearance of cancer symptoms or treatment side effects; 3) diagnosis by a traditional healer;
- Partnering with traditional healers, who are important gatekeepers in the community, may lead to better community outreach, increased biomedical referrals and greater uptake of biomedical treatment for cancer, resulting in better illness outcomes;
- The importance of understanding the public health impact of how previously-implemented educational campaigns (e.g regarding HIV) may affect current knowledge uptake regarding educational campaigns about cancer.

## Competing interests

The authors declare no competing interest.
